# HYAL3 as a therapeutic target for pulmonary arterial Hypertension–Cardiomyopathy comorbidity: an integrative analysis combined with machine learning and SHAP value interpretation

**DOI:** 10.3389/fphar.2026.1759555

**Published:** 2026-04-10

**Authors:** Jianchuan Hu, Wanqun Zhang, Qiurong Zeng, Min Yang, Lili Zhang, Yunhu Xu

**Affiliations:** 1 General Practice Department, Chongqing University Fuling Hospital, Chongqing University, Chongqing, China; 2 Hepatology Department, Chongqing University Fuling Hospital, Chongqing University, Chongqing, China

**Keywords:** HYAL3, machine learning, pulmonary arterial hypertension-cardiomyopathy comorbidity, SHAP analysis, virtual screening

## Abstract

**Background:**

This study aimed to elucidate the molecular mechanisms driving the comorbidity of pulmonary arterial hypertension and cardiomyopathy (PAH-CMP), identify key pathogenic genes, and explore potential therapeutic agents targeting these genes.

**Methods:**

Common targets for PAH and CMP were retrieved from public databases. Weighted gene co-expression network analysis (WGCNA) and multiple machine learning algorithms were employed to screen diagnostic hub genes. Upstream regulatory networks were constructed to explore molecular interactions. Key findings were validated *in vitro* using hypoxia-induced human pulmonary artery endothelial cells (HPAECs) and angiotensin II-induced H9C2 cardiomyocytes. Finally, molecular docking and dynamics simulations were performed to identify drug candidates.

**Results:**

In total, 14,403 genes common to PAH and CMP were identified. WGCNA identified a key module that was strongly correlated with PAH-CMP. Integrative machine learning highlighted four hub genes, HYAL3, ADIPOQ, ZNF852, and SCD, all of which showed excellent diagnostic value for PAH-CMP comorbidity, with an area under curve exceeding 0.9. Regulatory network analysis revealed potential upstream transcription factors, including FOXL1, FOXC1, and PPARG. The SHAP analysis demonstrated the strong contribution of these genes to the model, with HYAL3 exhibiting the highest feature importance. Experimental validation confirmed that HYAL3 was significantly upregulated in both the PAH endothelial cell model and the CMP cardiomyocyte model, corroborating the bioinformatic predictions. Virtual screening identified flurbiprofen as a high-affinity binder for HYAL3, and molecular dynamics simulations demonstrated stable binding between them.

**Conclusion:**

This study systematically identified and experimentally validated HYAL3, ADIPOQ, ZNF852, and SCD as core genes in PAH-CMP comorbidity and proposed flurbiprofen as a candidate therapeutic agent targeting HYAL3. Our findings provide mechanistic insights and a potential pharmacological strategy for the treatment of PAH-CMP.

## Introduction

Pulmonary arterial hypertension (PAH) is a progressive and life-threatening cardiopulmonary disorder characterized by sustained elevation of pulmonary vascular resistance, resulting in right ventricular pressure overload and eventual failure (Cuthbertson et al., 2023; Thompson et al., 2017; Wang et al., 2023; Ruopp et al., 2022). The pathogenesis of this disease involves pulmonary endothelial dysfunction, abnormal vascular remodeling, and smooth muscle proliferation (Zolty et al., 2020). Despite its low incidence, PAH is associated with a high mortality rate. Cardiomyopathies (CMP), including dilated and hypertrophic subtypes, constitute a heterogeneous group of myocardial disorders defined by structural and functional impairment, and represent important causes of heart failure and sudden cardiac death (Covella et al., 2017). Improved detection using advanced cardiac imaging and genetic testing has contributed to a rising global disease burden.

Clinically, PAH and cardiomyopathy often co-occur and influence each other through interconnected mechanisms (Seferović et al., 2019). Myocardial involvement is more frequently observed in patients with PAH than in the general population (Tello et al., 2019). Chronic pressure overload leads to right ventricular hypertrophy, fibrosis, and dysfunction, which can subsequently compromise left ventricular function through ventricular interdependence (Lambert et al., 2021). In contrast, cardiomyopathy related to left heart disease often contributes to PAH through elevated pulmonary venous pressure and reactive pulmonary vascular remodeling, creating a self-perpetuating pathological cycle (Maron et al., 2023; Maeder et al., 2022). Furthermore, recent insights highlight the complex hemodynamic consequences of left-ventricle pathologies on pulmonary venous resistance and right ventricular afterload, emphasizing the need to understand this bidirectional cardiopulmonary axis (Maron et al., 2024). This interdependent relationship complicates clinical management and predicts a worse prognosis. Growing evidence suggests that inflammatory responses, immune dysregulation, and epigenetic alterations are involved in both disease processes (Estupiñán-Moreno et al., 2022); however, the precise molecular mechanisms underlying their co-occurrence remain poorly understood.

Currently available PAH-specific therapies, such as endothelin receptor antagonists and phosphodiesterase-5 inhibitors, primarily act by inducing pulmonary vasodilation, but have limited impact on reversing myocardial remodeling or fibrosis (Hsieh et al., 2022; Lareo et al., 2021). In addition to these, prostacyclin analogs remain a cornerstone and one of the most promising options in managing PH. More importantly, emerging therapies such as activin signaling inhibitors (e.g., Sotatercept) have demonstrated the potential to not only manage symptoms but actively treat the underlying vascular remodeling pathologies that cause PH (Weinstein et al., 2024). Conventional heart failure drugs, including diuretics and beta-blockers, require cautious application in patients with combined PAH and cardiomyopathy because of the potential risk of worsening right ventricular function or systemic hemodynamics (Zheng et al., 2020; Benza et al., 2022). These therapeutic challenges highlight the urgent need for novel agents that can concurrently target pulmonary vascular and cardiac pathologies.

In this study, we integrated multi-omics data with bioinformatics approaches to identify HYAL3 as a key mediator of PAH-CMP. Furthermore, we propose flurbiprofen as a promising therapeutic agent for this target. Our results provide new mechanistic insights into cardiopulmonary disease interactions and establish a rationale for developing HYAL3 -directed therapies with dual therapeutic benefits in this complex patient population.

## Methods

### Disease-related targets for PAH and CMP

To comprehensively identify disease-associated genes for PAH and CMP, we searched three major databases: GeneCards (Stelzer et al., 2016), Online Mendelian Inheritance in Man (OMIM) (Hamosh et al., 2004), and Comparative Toxicogenomics Database (CTD). The search keywords used were “pulmonary arterial hypertension” and “cardiomyopathy”. After integrating the results from all sources, we removed duplicates to finalize the shared gene sets of PAH-CMP for subsequent analyses.

### Data acquisition and preprocessing

Transcriptomic profiles were obtained from the Gene Expression Omnibus (GEO) database (https://www.ncbi.nlm.nih.gov/geo/). For pulmonary arterial hypertension (PAH), the dataset GSE130391 was utilized. For cardiomyopathy (CMP), datasets GSE1145 and GSE19303 were downloaded. Raw data were processed and log_2_ transformed. For datasets requiring integration, batch effects were corrected using the empirical Bayes framework implemented in the “sva” (Surrogate Variable Analysis) R package. Genes with low expression levels across all samples were filtered out, and the normalized expression matrices were subsequently utilized for downstream Weighted Gene Co-expression Network Analysis (WGCNA) and machine learning evaluations.

### Weighted gene co-expression network analysis (WGCNA)

During data preprocessing, genes with low expression levels were filtered out, defined as genes with counts per million (CPM) < 1 in more than 50% of the samples. Crucially, to biologically anchor our transcriptomic analysis to the previously identified disease mechanisms, the preprocessed GEO expression matrix was then intersected with the 14,403 shared targets of PAH and CMP. Only the expression profiles of these intersecting genes were retained for the subsequent Weighted Gene Co-expression Network Analysis (WGCNA). We employed the R package “WGCNA” (Langfelder et al., 2008) to identify biologically significant co-expression modules and explore their association with disease. Specifically, a scale-free co-expression network was constructed by evaluating various β-parameters, from which a value of 20 was determined to be optimal. Co-expression modules were then identified using the dynamic tree-cut algorithm, with a minimum module size of 50 genes. Subsequently, highly similar modules were merged using dynamic hybrid merging, with a merging threshold of 0.05. Finally, modules significantly correlated with clinical traits were identified based on the Pearson correlation between their module eigengenes (MEs) and traits.

### Kyoto encyclopedia of genes and genomes (KEGG) pathways and gene ontology (GO) enrichment analysis

To elucidate the biological functions and signaling pathways associated with PAH-CMP comorbidity, the genes within the key co-expression module (the gray module) identified via WGCNA underwent enrichment analysis of KEGG pathways and GO terms using the “clusterProfiler” R package. The enrichment results were visualized using the “enrichplot” and “ggplot2” packages, with the “org.Hs.eg.db” package employed for gene identifier mapping.

### Machine learning algorithms

We employed three distinct machine learning algorithms to identify potential targets for PAH-CMP comorbidity. First, we performed feature selection using Least Absolute Shrinkage and Selection Operator (LASSO) regression with 10-fold cross-validation via the “glmnet” R package (Li et al., 2022). Subsequently, we applied a Random Forest (RF) algorithm using the “randomForest” package, which operates by aggregating predictions from multiple decision trees (Little et al., 2022). Finally, we utilized a Support Vector Machine (SVM) classifier that finds an optimal hyperplane for sample separation in a high-dimensional space (Hu et al., 2011). The intersecting genes identified by all the three algorithms were identified as candidate targets.

### Receiver operating characteristic curve (ROC) analysis of candidate genes

To validate our findings, T-tests were conducted to identify intergroup differences, with a *P*-value <0.05 defined as statistically significant. Subsequently, ROC curves were constructed and the area under the curve (AUC) was calculated via the “pROC” package to examine the diagnostic potential of candidate targets, where an AUC >0.7 was considered ideal. Furthermore, a multi-gene diagnostic model was developed using logistic regression analysis, and the performances of the single- and multi-gene models were compared by analyzing their ROC curves.

### Gene set enrichment analysis

Gene set enrichment analysis (GSEA, v4.1.0) was performed on the four candidate genes, based on their expression profiles, to identify associated biological processes and pathways.

### The regulatory network analysis

To construct the miRNA-mRNA-transcription factor (TF) regulatory network, miRNA-mRNA interaction data were obtained from TarBase and TF-mRNA interactions were obtained from JASPAR using NetworkAnalyst 3.0 (https://www.networkanalyst.ca/). The resulting set of mRNAs, miRNAs, and TFs was used to build and visualize the integrated network in Cytoscape V3.9.0.

### Explanation of machine learning models

To interpret the decision-making process of our diagnostic model and quantify the specific contribution of each core gene, we applied the SHapley Additive exPlanations (SHAP) (Lundberg and Lee, 2017) method directly to the Random Forest (RF) model developed during the initial feature selection phase. SHAP employs a game-theoretic approach that aggregates the local contributions of individual features to explain a model’s behavior on a global scale. By calculating SHAP values, we were able to robustly rank the overall feature importance of the four hub genes, thereby identifying the primary molecular driver for subsequent virtual screening. To prevent overfitting and ensure robust evaluation of this specific RF model, a 5-fold cross-validation framework was applied. During the training phase, automated hyperparameter tuning (utilizing a search length of 5) was conducted to optimize the RF model’s predictive performance. The model was strictly optimized and selected based on the criterion of achieving the highest Area Under the Curve (AUC) upon cross-validation. The detailed configuration of the optimized hyperparameters for this final RF model is provided in Supplementary Table S1.

### Virtual screening and molecular docking

We performed virtual screening of an FDA-approved drug library to identify potential therapeutics for PAH-CMP comorbidities via drug repurposing. The target protein structure was sourced from UniProt and FDA-approved drug structures were retrieved from the ZINC database (Irwin et al., 2005). Ligand preparation was conducted using the LigPrep module in Schrödinger Maestro 12.8, which included hydrogen addition, ionization state generation at pH 7.0 ± 2.0, and energy minimization with the OPLS2005 force field to yield optimized 3D geometries. For HYAL3, a homology model (SWISS-MODEL: O43820_22-412:2pe4.1. A) was obtained and prepared using AutoDock Tools: removal of water molecules, addition of polar hydrogens, charge assignment, and conversion to the PDBQT format. Molecular docking of 1,531 compounds was carried out with AutoDock Vina-GPU 2.1 (Eberhardt et al., 2021; Morris et al., 2009) using a grid box (18 Å × 16 Å × 20 Å) centered on the active site of the protein. The compounds were ranked by binding affinity (kcal/mol), with lower values indicating stronger binding. The top five scoring complexes were visualized using Discovery Studio 2019.

### Molecular dynamics (MD)

MD simulations were performed using the GROMACS 2025.1. Flurbiprofen was parameterized with the GAFF force field using the Sobtop program (v1.0) and subsequently incorporated into the system topology. The proteins were described using the AMBER99SB-ILDN force field. The complex was solvated in a cubic water box of TIP3P and neutralized with Na + ions. The system was first subjected to energy minimization using the steepest descent algorithm, followed by 50,000 steps of NVT equilibration and 50,000 steps of NPT equilibration. A production MD simulation was then run for 50,000,000 steps with a 2-fs timestep, generating a 100-ns simulation time. The root-mean-square deviation (RMSD), root-mean-square fluctuation (RMSF), radius of gyration (Rg), and solvent-accessible surface area (SASA) were computed.

### Cell culture

HPAECs were obtained from Procell Life Science & Technology (Wuhan, China) and cultured in medium (endothelial cell medium (ECM) for HPAECs, Sciencell, 1001, CA, United States) containing 10% fetal bovine serum and 1% penicillin-streptomycin at 37 °C, 5% CO2, and 100% relative humidity. For the hypoxia exposure experiments, HPAECs were incubated in a Tri-Gas Incubator (Heal Force) in a water-saturated atmosphere containing 2% O2 and 5% CO2 for 48 h. The H9C2 cell line was purchased from Meilunbio and cultured in DMEM (PM150210, Pricella, China) supplemented with 10% fetal bovine serum (FBS, UT82901, TOCYTO, United States) and 1% penicillin/streptomycin solution (MA0110, Meilunbio, China) at 37 °C under 95% O2 and 5 %CO2. For the Ang II exposure assay, H9C2 cells were incubated in basal medium containing AngII (1 μmol/L) for 48 h. For the experiments involving flurbiprofen treatment, 10 μmol/L flurbiprofen was administered to HPAECs and H9C2 cells respectively during their hypoxic treatment and Ang II treatment. The cells were further detected after 48 h.

### Western blot analysis

The proteins were extracted from HPAECs and H9C2s by using RIPA (P0013B; Beyotime Biotechnology, Shanghai, China) buffer supplemented with PMSF (ST506; Beyotime Biotechnology, Shanghai, China). The 30 μg protein samples were fractionated by 10% SDS–PAGE, transferred onto nitrocellulose membranes, and subsequently blocked with 5% nonfat milk at room temperature for 1 h. Anti-HYAL3 antibody (1:1000, #822521, Zenbio, Chengdu, China), anti-beta Actin antibody (1:1000, #R380624, Zenbio, Chengdu, China), anti-GAPDH antibody (1:1000, #R380626, Zenbio, Chengdu, China), were used as primary antibodies and were incubated overnight at 4 °C, followed by incubation with appropriate horseradish peroxidase-conjugated secondary antibodies at room temperature for 1 h and enhanced chemiluminescent reagents imaging.

### Quantitative real-time polymerase chain reaction (qRT–PCR)

Total RNA was extracted from HPAECs and H9C2s with TRIzol reagent (Invitrogen, CA, United States) according to the manufacturer’s protocol. cDNA was synthesized from 2 μg of RNA using the Superscript First-Strand Complementary DNA Synthesis Kit (Abclonal, Wuhan, China). To measure the RNA expression levels, the cDNA products were quantified using SYBR Green real-time PCR (Abclonal, Wuhan, China) in a Bio-Rad, United States instrument. GAPDH was used as the internal control. The threshold cycle (Ct) was determined, and the data were analyzed using the 2^−ΔΔCT^ method. The sequences of the primers used are listed in Supplementary Table S2.

### CCK8 assay

HPAECs and H9C2s were cultured in 96-well plates at a density of 5000 cells/well and treated with different reagents. After incubating at 37 °C for an additional 48 h, 10 μL of CCK8 reagent were added to each well, which was subsequently incubated for 2–4 h at 37 °C, after which the absorbance at 450 nm was measured using a spectrophotometric microplate reader.

### Statistical analysis

All bioinformatics and statistical analyses were conducted using R software (version 4.2.2) and GraphPad Prism (version 9.0). Continuous variables from *in vitro* experiments were presented as the mean ± standard deviation (SD) from at least three independent biological replicates. For comparisons between two independent groups, a two-tailed Student’s t-test was employed. For multiple group comparisons, a One-way Analysis of Variance (ANOVA) followed by Tukey’s *post hoc* test was utilized to assess statistical significance. The diagnostic performance of machine learning models was evaluated using the AUC from ROC analyses. A *P*-value <0.05 was considered statistically significant (*P < 0.05, **P < 0.01, ***P < 0.001, ****P < 0.0001).

## Results

**FIGURE 1:**
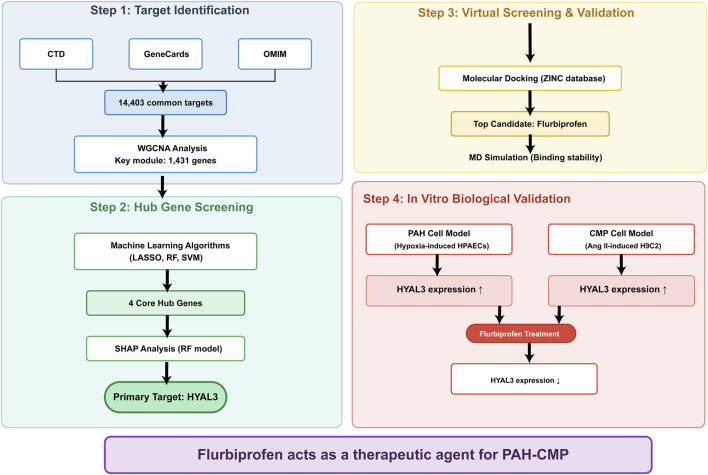
Flowchart of this study.

The flowchart for this study is shown in Figure 1.

### Screening and enrichment analysis of targets for PAH-CMP comorbidity

**FIGURE 2:**
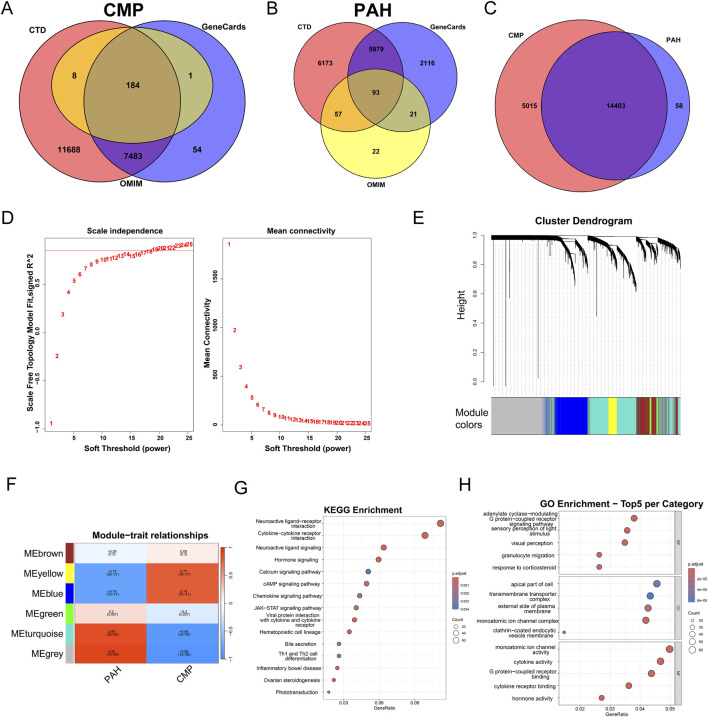
WGCNA analysis of the significant genes in CMP and PAH **(A,B)** Venn diagrams of CMP-**(A)** and PAH-**(B)** related genes from the CTD, GeneCards, and OMIM databases. **(C)** Venn diagrams of the CMP-PAH intersection genes. **(D)** Selection of the soft threshold in CMP and PAH. **(E)** Gene cluster dendrogram for CMP and PAH by dynamic tree cut algorithm **(F)** Heatmap of the relationships between CMP and PAH. **(G,H)** Gray module **(G)** KEGG and **(H)** GO enrichment analysis.

Potential target genes associated with both PAH and CMP were identified using the GeneCards, OMIM, and CTD databases (Figures 2A,B). A Venn diagram revealed 14403 genes common to both PAH and CMP (Figure 2C). To rigorously eliminate inherent false positives from this merged dataset and perform dimensionality reduction, we applied weighted gene co-expression network analysis (WGCNA) to empirical transcriptomic data to identify modules most strongly associated with PAH–CMP. During network construction, although a power of 19 reached the 0.85 threshold, a soft threshold of 20 was ultimately selected to achieve a more robust scale-free topology fit index (0.866) while maintaining suitable mean connectivity (Figure 2D). Figure 2E shows the clustering dendrogram of samples from the PAH and CMP groups. This analysis generated six color-coded co-expression modules (Figure 2F). Among them, the gray module, containing 1431 genes, was identified as the key module for subsequent analysis because of its high gene significance (r = 0.89, p = 1e-05).

To further investigate the biological relevance of the gray module, we performed KEGG pathway and GO functional enrichment analyses. The most significantly enriched KEGG pathway was “Neuractive ligand-receptor interaction.” GO analysis revealed that these genes were significantly enriched across biological process (BP), cellular component (CC), and molecular function (MF) categories. Notably, the most prominent BP term was “adenylate cyclase−modulating G protein−coupled receptor,” the leading CC term was “apical part of cell,” and the top MF term was “monoatomic ion channel activity” (Figures 2G,H).

### Selection of hub genes using machine learning

**FIGURE 3:**
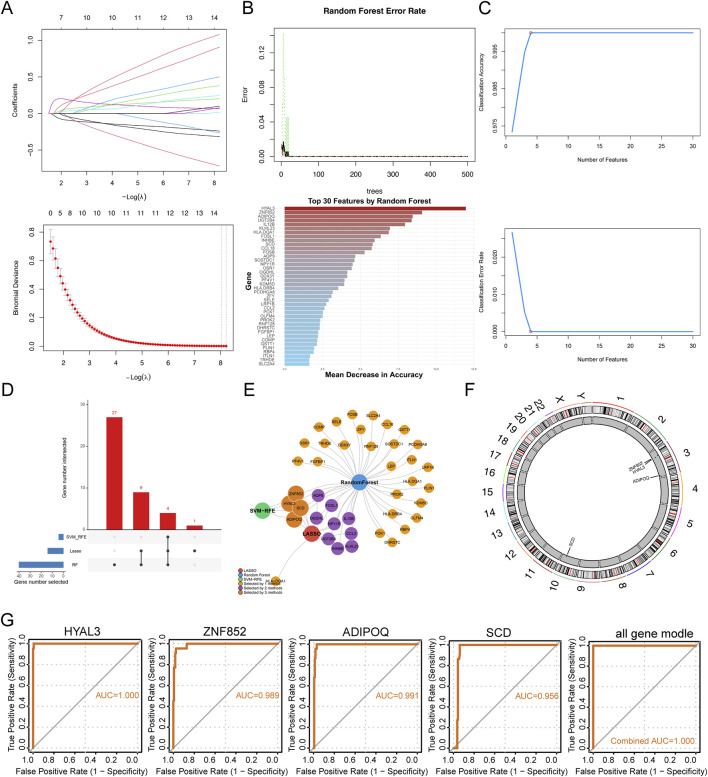
Identification of candidate biomarkers by machine learning **(A–C)** Key gene identification using three machine learning algorithms: **(A)** LASSO regression, **(B)** Random Forest (RF), and **(C)** Support Vector Machine (SVM), with respective parameter optimization and error rate shown. **(D)** Venn diagram of the intersection of genes identified by the three algorithms. **(E)** Visualization of gene names at the intersection of the three machine-learning methods. **(F)** Chromosomal localization of four core hub genes. **(G)** ROC curves of the four hub genes in CMP and PAH (GSE1145, GSE19303, and GSE130391 datasets), with corresponding AUC values.

We employed three machine learning methods, LASSO regression, RF, and SVM, to identify potential target genes associated with PAH-CMP comorbidity. LASSO regression selected 13 candidate markers (Figure 3A), while the RF algorithm identified 40 candidate genes based on importance scores (Figure 3B). The support vector machine approach identified four candidate genes through accuracy profiling and cross-validation error analysis (Figure 3C). Integration of the results from all three methods yielded four intersecting candidate genes: HYAL3, ADIPOQ, ZNF852, and SCD (Figures 3D,E). The locations of the candidate genes on the chromosomes are shown in Figure 3F. To assess the potential diagnostic value of these genes in PAH-CMP comorbidity, we performed an ROC analysis (Figure 3G). The results demonstrated that these markers exhibited strong predictive potential for the disease within the analyzed retrospective transcriptomic cohorts, both individually and in combination.

### GSEA results

**FIGURE 4:**
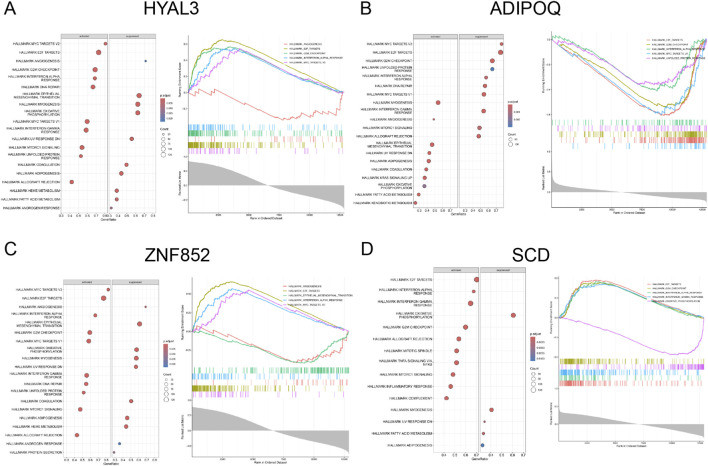
GSEA enrichment analysis of the four hub genes. **(A–D)** GSEA results of four hub genes. **(A)** HYAL3, **(B)** ADIPOQ, **(C)** ZNF852, and **(D)** SCD. Each subplot shows the enriched hallmark pathways and the corresponding enrichment metrics.

Gene set enrichment analysis (GSEA) was performed to identify biological processes and molecular functions associated with the four candidate genes (HYAL3, ADIPOQ, ZNF852, and SCD) in PAH-CMP comorbidity (Figure 4). The top five significantly enriched pathways were identified based on the expression levels of these genes. In both PAH (GSE130391) and CMP datasets (GSE1145, GSE19303), pathways including E2F targets, G2M checkpoint, interferon alpha response, and MYC target V2 were markedly downregulated (Figure 4). In contrast, the angiogenesis and epithelial-mesenchymal transition pathways were significantly upregulated.

### The regulatory network analysis

**FIGURE 5:**
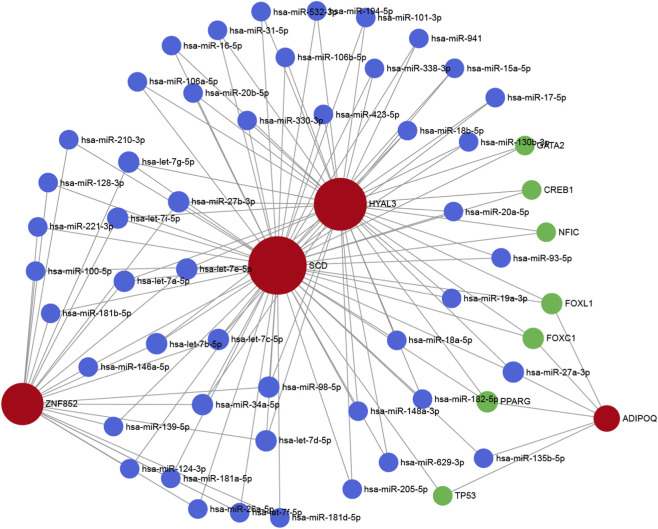
TFs-miRNA-mRNA interaction network of the four core genes The TF-miRNA-mRNA interaction network was centered on the four core mRNAs. Nodes represent transcription factors (TFs, green), microRNAs (miRNAs, blue), and four core mRNAs (red: HYAL3, ADIPOQ, ZNF852, SCD), with edges indicating regulatory interactions between molecules.

To elucidate the upstream regulatory mechanisms of the four candidate genes (HYAL3, ADIPOQ, ZNF852, and SCD) in PAH-CMP, we constructed an miRNA–mRNA–TF network (Figure 5). Through prediction analyses, we identified 47 miRNAs targeting these genes and 7 transcription factors (TFs) regulate their expression. The resulting integrative network comprised of four hub genes, 47 miRNAs, and seven TFs. Among these TFs, FOXL1, FOXC1, and PPARG were found to regulate HYAL3, ADIPOQ, and SCD. Previous studies have shown that loss of PPARG aggravates pulmonary arterial hypertension, whereas its upregulation attenuates vascular remodeling and PAH severity. Additionally, FOXC1 has been implicated in oxidative stress pathways, indicating its potential role in PAH, where oxidative stress is a key pathological mechanism. Collectively, these findings suggest that coordinated transcriptional and post-transcriptional regulation may regulate the roles of these hub genes in PAH-CMP.

### SHAP analysis results

**FIGURE 6:**
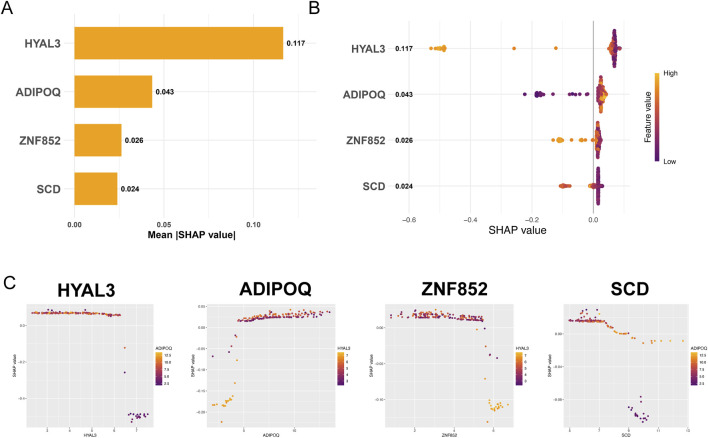
SHAP analysis for determining the most contributing hub gene based on the Random Forest model. **(A)** Bar plot of feature importance based on SHAP values, presenting the relative contribution of the four hub genes. **(B)** Beeswarm plot showing the distribution of the feature effects for each hub gene. **(C)** Feature dependency contribution map of the hub genes.

To interpret the decision-making process of the established Random Forest model and determine the individual contribution of the four core genes, we employed SHAP analysis (Figure 6). The overall feature importance is summarized in a bar chart, demonstrating that HYAL3 exhibited the highest SHAP value and greatest influence on the model output (Figure 6A). The distribution and mean impact of each gene across all samples were further illustrated in a beeswarm plot (Figure 6B). Dependence plots were utilized to reveal how individual gene expression drives the clinical outcome (Figure 6C). Collectively, these results highlighted HYAL3 as the primary molecular driver, providing a solid rationale for selecting it as the primary target for subsequent virtual screening.

### Virtual screening of the ZINC database targeting HYAL3

**FIGURE 7:**
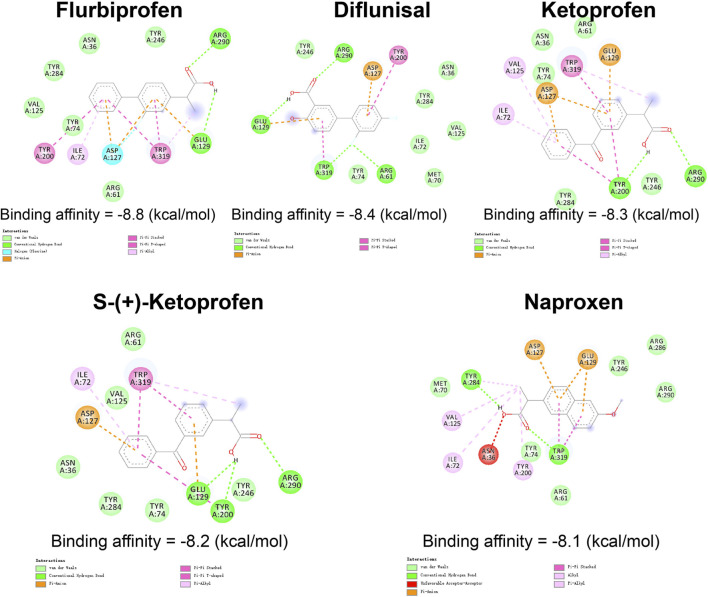
Virtual drug screening for the key gene HYAL3 Visualization of the top five drugs with the strongest binding affinities from virtual screening targeting HYAL3: Flurbiprofen (binding energy: 8.8 kcal/mol); Diflunisal (binding energy: 8.4 kcal/mol); Ketoprofen (binding energy: 8.3 kcal/mol); S-(+)-ketoprofen (binding energy: 8.2 kcal/mol); Naproxen (binding energy: 8.1 kcal/mol). All subplots depict drug-HYAL3 binding modes, with key interacting amino acids and labelled binding energy values.

Based on the identification of HYAL3 as a key contributor to PAH-CMP comorbidities, we developed a virtual screening workflow to target HYAL3 using the ZINC database. We assessed the binding affinity of over 1,500 commercially available FDA-approved compounds to HYAL3 through molecular docking with AutoDock Vina (Figure 7). The top five compounds selected for their strong binding affinities were Flurbiprofen (−8.8 kcal/mol), Diflunisal (−8.4 kcal/mol), Ketoprofen (−8.3 kcal/mol), S-(+)-ketoprofen (−8.2 kcal/mol), and Naproxen (−8.1 kcal/mol). The stable interactions between the top-ranked compounds and HYAL3 highlight their potential as therapeutic agents for PAH-CMP treatment.

### MD analysis of flurbiprofen binding to HYAL3

**FIGURE 8:**
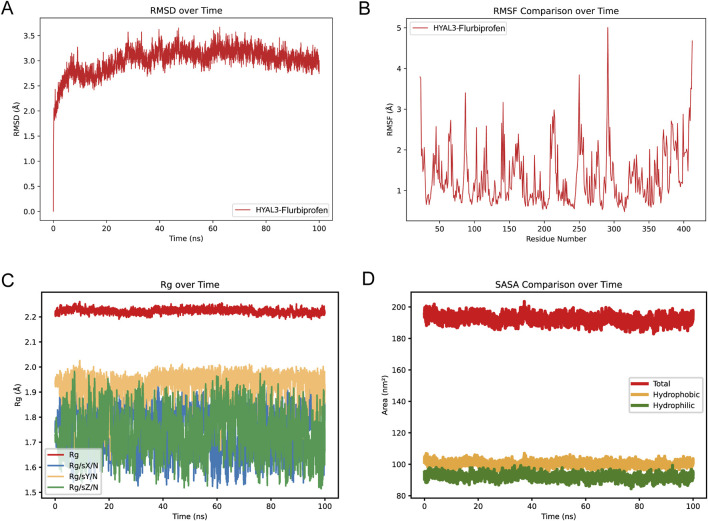
Molecular dynamics (MD) simulation of HYAL3-Flurbiprofen complex **(A)** Root mean square deviation (RMSD) over time for the HYAL3-Flurbiprofen complex. **(B)** Root mean square fluctuation (RMSF) comparison over time showing residue flexibility. **(C)** Radius of gyration (Rg) over time, depicting the compactness of the complex. **(D)** Comparison of Solvent-accessible surface area (SASA) over time, including total, hydrophobic, and hydrophilic SASA.

To evaluate the binding stability between HYAL3 and flurbiprofen, we performed a 100 ns MD simulation of the HYAL3–flurbiprofen complex in aqueous solution using GROMACS. RMSD analysis indicated that the system reached equilibrium after approximately 30 ns, with fluctuations significantly decreasing and remaining stable thereafter, suggesting the formation of a stable protein–ligand binding conformation (Figure 8A). The RMSF profile suggested fluctuations in amino acid residues, with lower values indicating higher structural stability in the corresponding regions (Figure 8B). Moreover, the radius of analysis showed only minor variations throughout the simulation, indicating a compact and highly stable complex structure (Figure 8C). Our findings showed that the HYAL3-flurbiprofen complex showed no large fluctuations in SASA values, indicating that the complex was stable (Figure 8D). Collectively, these results demonstrated that a stable interaction was formed between HYAL3 and flurbiprofen.

### HYAL3 upregulated in both the PAH endothelial cell model and CMP cardiomyocyte model

**FIGURE 9:**
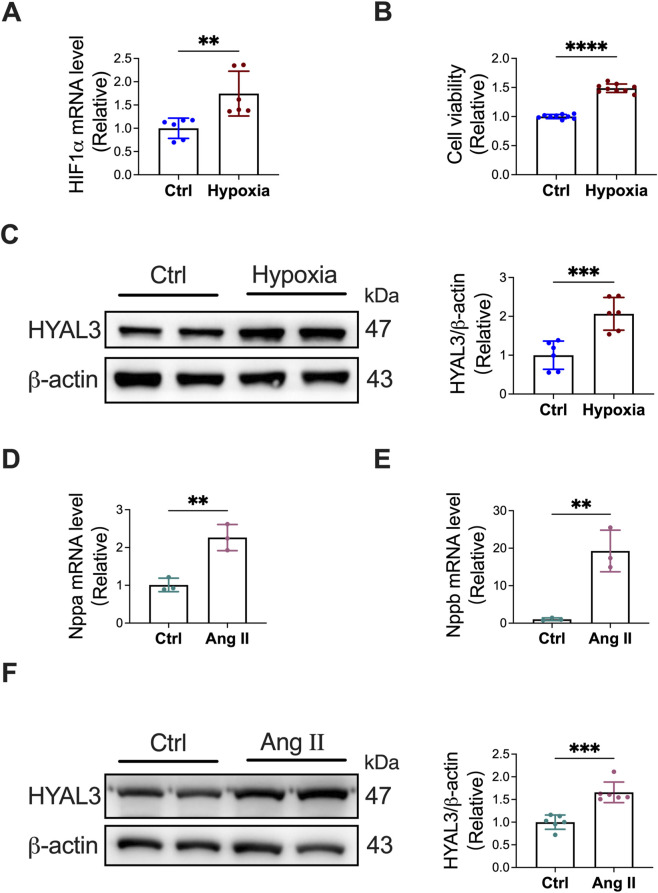
Validation of HYAL3 upregulation in PAH endothelial cell and CMP cardiomyocyte models. **(A–C)** Establishment of the PAH cell model in human pulmonary artery endothelial cells (HPAECs) induced by hypoxia for 48 h. **(A)** Relative mRNA expression of HIF-1α determined by qRT-PCR. **(B)** Cell viability assessed by CCK-8 assay. **(C)** Representative Western blot images and quantitative analysis of HYAL3 protein expression in HPAECs. **(D–F)** Establishment of the CMP cell model in H9C2 cardiomyocytes induced by Angiotensin II (Ang II) for 48 h **(D,E)** Relative mRNA expression levels of Nppa **(D)** and Nppb **(E)** determined by qRT-PCR. **(F)** Representative Western blot images and quantitative analysis of HYAL3 protein expression in H9C2 cells. Data are presented as mean ± SD. **P < 0.01, ***P < 0.001, ****P < 0.0001 vs. Ctrl group.

To validate the key findings from bioinformatics analyses, we first constructed cell models of pulmonary arterial hypertension (PAH) and cardiomyopathy (CMP) respectively, and then detected the expression of the hub gene HYAL3 in both models. For the PAH cell model construction, human pulmonary artery endothelial cells (HPAECs) were subjected to hypoxic stimulation for 48 h, with normoxic cultured cells as the control group. Quantitative real-time polymerase chain reaction (qRT-PCR) was performed to detect the expression of hypoxia-inducible factor 1α (HIF-1α), a classic marker of hypoxic response and PAH endothelial cell activation. Cell Counting Kit-8 (CCK-8) assay was used to evaluate the proliferation capacity of HPAECs. As shown in Figures 9A,B, compared with the normoxic control group, the mRNA expression level of HIF-1α in HPAECs was significantly upregulated after 48 h of hypoxia (P < 0.01), and the cell proliferation activity was also significantly enhanced (P < 0.01), confirming the successful establishment of the PAH endothelial cell model. Subsequent Western blot (WB) analysis was conducted to detect the protein expression of HYAL3 in the PAH model. The results showed that the protein level of HYAL3 in hypoxic HPAECs was significantly higher than that in the control group (P < 0.01), indicating that HYAL3 was upregulated in the PAH cell model (Figure 9C).

For the CMP cell model construction, H9C2 cardiomyocytes were induced with angiotensin II (Ang II) to establish a cardiomyopathy model, with untreated cardiomyocytes as the control group. The expression levels of natriuretic peptide A (Nppa) and natriuretic peptide B (Nppb), two well-recognized pathogenic factors and diagnostic markers of cardiomyopathy, were detected by qRT-PCR to verify the model validity. As illustrated in Figures 9D,E, compared with the control group, the mRNA expressions of Nppa and Nppb in H9C2 cells were significantly upregulated after 48 h of Ang II treatment (both P < 0.01), confirming the successful establishment of the CMP cell model. WB analysis was further performed to detect the protein expression of HYAL3 in the CMP model, and the results showed that the protein level of HYAL3 in Ang II-induced H9C2 cells was significantly increased compared with the control group (P < 0.01) (Figure 9F). Collectively, the above cell experimental results demonstrated that HYAL3 was significantly upregulated in both PAH endothelial cell model and CMP cardiomyocyte model.

### Flurbiprofen as a potential targeted inhibitor of HYAL3

Prior virtual screening studies identified flurbiprofen as a high-affinity binder for HYAL3, and subsequent MD simulations further confirmed the stable binding interaction between flurbiprofen and HYAL3, supporting flurbiprofen as a potential targeted inhibitor of HYAL3. To verify the biological effect of flurbiprofen on HYAL3 and its regulatory role in PAH and CMP pathological processes, we performed flurbiprofen intervention experiments on the established PAH endothelial cell model (hypoxia-induced HPAECs) and CMP cardiomyocyte model (Ang II-induced H9C2 cells), with corresponding model groups (without flurbiprofen treatment) and normal control groups set as references.

**FIGURE 10:**
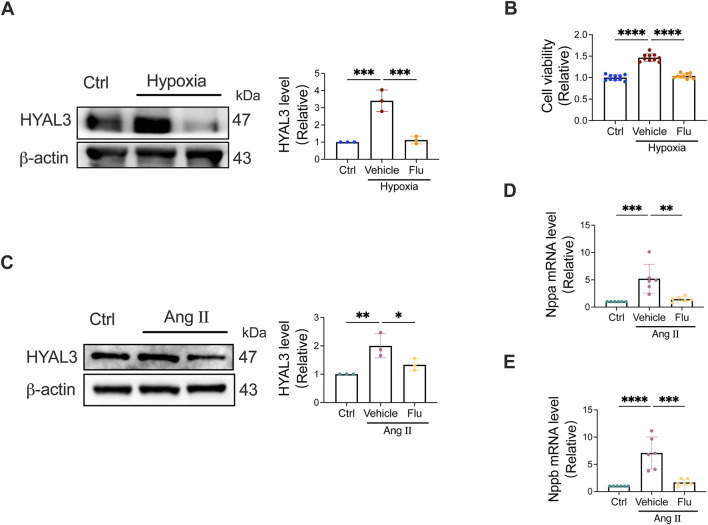
Flurbiprofen inhibits HYAL3 expression and rescues pathological phenotypes in PAH and CMP cell models. **(A,B)** Effects of flurbiprofen on the PAH endothelial cell model. HPAECs were exposed to hypoxia and treated with flurbiprofen (Flu, 10 μmol/L) or vehicle for 48 h. **(A)** Representative Western blot images and quantitative analysis of HYAL3 protein expression. **(B)** Cell viability assessed by CCK-8 assay. **(C–E)** Effects of flurbiprofen on the CMP cardiomyocyte model. H9C2 cells were induced by Angiotensin II (Ang II) and treated with flurbiprofen (Flu, 10 μmol/L) or vehicle for 48 h. **(C)** Representative Western blot images and quantitative analysis of HYAL3 protein expression. **(D,E)** Relative mRNA expression levels of Nppa **(D)** and Nppb **(E)** determined by qRT-PCR. Data are presented as mean ± SD. *P < 0.05, **P < 0.01, ***P < 0.001, ****P < 0.0001.

In the PAH endothelial cell model, HPAECs were divided into three groups: normoxic control group, hypoxia-induced PAH model group, and hypoxia + flurbiprofen (10 μmol/L) treatment group. After 48 h of concurrent treatment, Western blot (WB) analysis was performed to detect the protein expression of HYAL3. The results showed that compared with the normoxic control group, the protein level of HYAL3 in the PAH model group was significantly upregulated (P < 0.01); while flurbiprofen intervention significantly downregulated the hypoxia-induced high expression of HYAL3 in HPAECs (P < 0.01), confirming the inhibitory effect of flurbiprofen on HYAL3 in PAH-related pathological conditions (Figure 10A). Additionally, CCK-8 assay was used to evaluate the effect of flurbiprofen on hypoxia-induced HPAEC proliferation. Consistent with the previous model validation results, the proliferation activity of HPAECs in the PAH model group was significantly higher than that in the control group (P < 0.01); flurbiprofen treatment effectively reversed the enhanced proliferation of HPAECs induced by hypoxia, and the cell proliferation activity was significantly reduced compared with the model group (P < 0.01), indicating that flurbiprofen could improve the abnormal proliferation phenotype of endothelial cells in the PAH model by inhibiting HYAL3 (Figure 10B).

In the CMP cardiomyocyte model, H9C2 cells were divided into three groups: normal control group, Ang II-induced CMP model group, and Ang II + flurbiprofen (10 μmol/L) treatment group. After 48 h of co-incubation, WB analysis was conducted to detect the protein expression of HYAL3. The results showed that the protein level of HYAL3 in the CMP model group was significantly higher than that in the normal control group (P < 0.01); flurbiprofen intervention significantly suppressed the Ang II-induced upregulation of HYAL3 in H9C2 cells (P < 0.05), further confirming the specific inhibitory effect of flurbiprofen on HYAL3 in CMP-related pathological environments (Figure 10C). To explore the regulatory effect of flurbiprofen on the key pathogenic phenotypes of CMP, qRT-PCR was used to detect the mRNA expression levels of Nppa and Nppb (core pathogenic genes of CMP) in each group. The results showed that compared with the normal control group, the mRNA expressions of Nppa and Nppb in the CMP model group were significantly upregulated (both P < 0.01); while flurbiprofen treatment significantly downregulated the Ang II-induced high expression of Nppa and Nppb (both P < 0.01), suggesting that flurbiprofen could alleviate the pathological state of the CMP model by inhibiting HYAL3 (Figures 10D,E).

Collectively, these results demonstrated that flurbiprofen, as a high-affinity binder of HYAL3, could effectively inhibit the abnormal upregulation of HYAL3 in both PAH endothelial cell model and CMP cardiomyocyte model. Furthermore, flurbiprofen could reverse the pathological phenotypes of the two models (abnormal proliferation of PAH endothelial cells, high expression of CMP pathogenic genes Nppa and Nppb) by targeting HYAL3.

## Discussion

The molecular basis of PAH and CMP remains poorly understood. Here, we applied a comprehensive bioinformatics and machine learning approach to identify four hub genes, including HYAL3, ADIPOQ, ZNF852, and SCD, which are central to the disease process. These genes showed strong diagnostic performance, supporting their use as a combined molecular signature. The SHAP analysis precisely quantified the individual contribution of each feature, with HYAL3 emerging as the most influential predictor. Therefore, we targeted HYAL3 for virtual screening, identifying flurbiprofen as the top high-affinity binder, a result confirmed with MD simulations. Methodologically, our unbiased co-expression network analysis offers distinct advantages over traditional literature-based reviews. While reviews primarily reiterate established pathways (e.g., endothelin, nitric oxide), our computational pipeline successfully recovers these known regulators while simultaneously uncovering novel, critical targets like HYAL3, demonstrating its superior robustness in biomarker discovery.

Our analysis identified 14403 common targets shared in PAH-CMP using multiple public databases, including GeneCards, OMIM, and CTD. Weighted gene co-expression network analysis (WGCNA) was then applied to transcriptomic data from the PAH and CMP datasets in GEO, identifying key modules strongly correlated with disease phenotypes. This initial step narrowed down the candidate gene pool from tens of thousands to a focused set of highly relevant genes. Three machine learning algorithms were integrated to further refine the candidate genes and identify four hub genes: HYAL3, ADIPOQ, ZNF852, and SCD. SHAP analysis was critical for interpreting this integrated model, as it quantified the specific contribution of each feature to the predictive output, with HYAL3 emerging as the most influential molecular driver.

HYAL3 encodes a hyaluronidase responsible for degrading hyaluronic acid (HA) within the extracellular matrix (Atmuri et al., 2008). The intact endothelial glycocalyx, an essential HA-rich structural layer, is critical for maintaining pulmonary vascular homeostasis. As demonstrated in our *in vitro* models, the abnormal upregulation of HYAL3 promotes excessive degradation of this protective glycocalyx. This continuous degradation disrupts endothelial integrity, thereby exacerbating pathological mechanotransduction and driving the vascular remodeling that characterizes PAH and cardiomyopathies. Consequently, targeted inhibition of HYAL3 hyperactivity presents a viable pharmacological strategy to preserve the endothelial glycocalyx and mitigate the progression of PAH-CMP comorbidity.

We constructed a regulatory network involving 30 transcription factors (TFs) and 47 miRNAs to elucidate the upstream regulatory mechanisms of the core genes. The network revealed that FOXL1, FOXC1, and PPARG target HYAL3, ADIPOQ, and SCD, pointing toward profound metabolic and transcriptomic dysregulation. ADIPOQ is a cardioprotective adipokine that acts to inhibit smooth muscle cell proliferation and mitigate pulmonary atherosclerosis-like lesions. Its upstream regulator, PPARG, drives lipid metabolism and interacts with TGFB1 and BMP2 signaling to attenuate vascular remodeling (Calvier et al., 2019; Calvier et al., 2017). The suppression of this ADIPOQ-PPARG axis under chronic hypoxia likely drives pathological lipid deposition. Additionally, aberrant activity of SCD, a key enzyme in fatty acid metabolism, can induce lipotoxicity and mitochondrial dysfunction during right ventricular pressure overload.

Furthermore, upstream TFs such as FOXC1, FOXL1, and the zinc finger protein ZNF852 orchestrate extensive transcriptomic reprogramming. FOXC1 directly regulates genes involved in oxidative stress metabolism (Xia et al., 2019; Jia et al., 2024). In the hypoxic PAH environment, FOXC1 and FOXL1 mediate fundamental phenotypic switching, driving extensive gene transcription changes required for processes such as endothelial-to-mesenchymal transition (EndMT). This aligns with our GSEA results, which revealed a significant upregulation of the EMT pathway. Recent literature highlights the critical role of ROS-driven pathways and GPX4-mediated metabolic homeostasis in mediating EndMT and vascular remodeling (Zhang et al., 2025; Ma et al., 2025). In this context, the transcriptional hub identified in our study may interface with broader immune-metabolic networks and ferroptosis pathways, which are increasingly recognized as crucial pathological drivers and emerging therapeutic frontiers in pulmonary vascular diseases (He et al., 2025; Phan et al., 2026).

We performed a structure-based virtual screening of over 1,500 approved drugs from the ZINC FDA database. Flurbiprofen is a traditional nonsteroidal anti-inflammatory drug (NSAID), which emerged as the top candidate based on its optimal binding energy (−8.8 kcal/mol) to the HYAL3 active site. Flurbiprofen is clinically used long-term for conditions such as arthritis and pain, primarily by inhibiting cyclooxygenase (COX) and reducing prostaglandin synthesis (Fukumoto et al., 2018). Our subsequent 100 ns MD simulations confirmed that the binding conformation of flurbiprofen with HYAL3 was highly stable, with the complex RMSD reaching equilibrium after 30 ns and maintaining minimal fluctuations. This finding suggests that flurbiprofen may exhibit a novel mechanism of action independent of COX inhibition by directly targeting HYAL3, potentially stabilizing its expression and restoring its activity. However, it is critical to acknowledge the pharmacological challenges and safety profiles associated with directly repurposing an NSAID for PAH-CMP comorbidities. As a non-selective COX inhibitor, flurbiprofen carries well-documented cardiovascular risks, including fluid retention and blood pressure elevation, which could be highly detrimental to patients with existing heart failure and PAH. To mitigate these systemic risks while harnessing its HYAL3-targeting potential, future therapeutic developments must address these pharmacokinetic limitations. Strategies should focus on designing structural derivatives of flurbiprofen that specifically lack COX-inhibitory activity but retain high binding affinity for HYAL3. Furthermore, employing targeted drug delivery systems, such as advanced controlled-release formulations or inhaled nanoparticles, could maximize local pulmonary and cardiac therapeutic efficacy while minimizing systemic adverse effects. By overcoming these translational hurdles, flurbiprofen provides a solid structural rationale and a promising pharmacological starting point for treating PAH-CMP.

Despite these promising findings, our study presents several limitations that warrant future investigation. First, the identification of hub genes and the evaluation of their diagnostic utility (high AUC values) were primarily based on gene co-expression networks from retrospective transcriptomic cohorts. This approach inherently overlooks crucial post-transcriptional mechanisms—including translation efficiency, protein stability, and dynamic cellular metabolic changes—and necessitates prospective clinical trials in independent, large-scale patient populations before these biomarkers can be confidently translated into clinical workflows. Second, the miRNA-mRNA-TF regulatory network presented here is fundamentally predictive. Direct functional interactions, such as the regulation of HYAL3 by FOXL1, FOXC1, or PPARG, remain speculative and require rigorous experimental validations utilizing ChIP-seq, luciferase reporter assays, and targeted siRNA knockdown. Finally, while our *in vitro* HPAEC and H9C2 models successfully validated the predictions regarding HYAL3 upregulation, these experiments were conducted under static monoculture conditions. Static models lack continuous fluid shear stress and cannot fully recapitulate the complex, multi-organ hemodynamics and sustained mechanical stress underlying PAH-CMP comorbidity *in vivo*. To address these limitations, future studies should incorporate microfluidic dynamic culture systems, proteomic analyses, *in vivo* rodent models (e.g., SU5416/hypoxia models with secondary right-to-left ventricular crosstalk), and primary patient-derived tissues to comprehensively enhance translational relevance.

## Conclusion

By integrating multi-omics data with network, machine learning, and SHAP analyses, this study identified HYAL3 as a pivotal molecule in PAH-CMP comorbidity and elucidated its upstream regulatory network. Furthermore, we repositioned the anti-inflammatory drug flurbiprofen as a potential therapeutic agent targeting HYAL3. Thus, our study provides both novel mechanistic insights and a mature candidate therapeutic strategy for this complex condition.

## Data Availability

The raw data supporting the conclusions of this article will be made available by the authors, without undue reservation.
