# Supplementary material for: HYAL3 as a therapeutic target for pulmonary arterial Hypertension–Cardiomyopathy comorbidity: an integrative analysis combined with machine learning and SHAP value interpretation
Source: Front Pharmacol. 2026 Apr 10;17:1759555. doi: 10.3389/fphar.2026.1759555 (PMC13106557; doi:10.3389/fphar.2026.1759555)
Supplement: Supplementary file 1 [file Table1.docx]

**Supplementary**

**Supplementary Table S1. Hyperparameter optimization details for the best-performing machine learning mode**

| **Hyperparameter** | **Description** | **Tuning Strategy** | **Optimal Configuration** |
| --- | --- | --- | --- |
| mtry | Number of variables randomly sampled as candidates at each split | Automated search (tuneLength = 5) | 2 |
| ntree | Number of trees to grow | Fixed default parameter | 500 |
| splitrule | Splitting rule | Fixed default parameter | Gini impurity |

**Supplementary Table S2. The sequences of the primers**

| Genes | Primer F | Primer R |
| --- | --- | --- |
| GAPDH-Human | TGTGGGCATCAATGGATTTGG | TGTGGGCATCAATGGATTTGG |
| HIF-1α-Human | TTCCCGACTAGGCCCATTC | CAGGTATTCAAGGTCCCATTTCA |
| GAPDH-Rat | GACATGCCGCCTGGAGAAAC | AGCCCAGGATGCCCTTTAGT |
| Nppa-Rat | CGATAGATCTGCCCTCTTGAAA | CTCCAATCCTGTCAATCCTACC |
| Nppb-Rat | TCCAGGAGAGACTTCGAAATTC | GCAAGTTTGTGCTGGAAGATAA |
